# Efficacy of indoor air purification in treating *Artemisia* (mugwort) pollen allergic rhinitis: study protocol for a randomised controlled trial

**DOI:** 10.1186/s12889-018-5678-0

**Published:** 2018-07-06

**Authors:** Qiao-yan Chen, Li Li, Li Zhang, Jin-han Mo, Zi-feng Yang, Xiao-lin Wei, Yun-ying Li, Ji-yan Xia, Xiao-bing Bai, Pei-fang Xie

**Affiliations:** 1 Guangdong Provincial Hospital of Chinese Medicine, The Second Affiliated Hospital, Guangzhou University of Chinese Medicine, 111 Dade Road, Guangzhou, 510120 China; 2 The First Hospital of Yulin, The Second Affiliated Hospital, Yanan University, 93 Yuxi Da Dao Road, Yulin, 719000 China; 3 Beijing Key Laboratory of Indoor Air Quality Evaluation and Control, Tsinghua University, 30 Shuangqing Road, Beijing, 100084 China; 4 State Key Laboratory of Respiratory Diseases, Guangzhou Institute of Respiratory Disease, National Clinical Centre of Respiratory Disease, The First Affiliated Hospital, Guangzhou Medical University, 151 Yanjiang Xi Road, Guangzhou, 510120 China; 5 University of Toronto, 27 King’s College Circle, Toronto, Ontario M5S 1A1 Canada

**Keywords:** Indoor air purification, Allergic rhinitis, *Artemisia*, Pollen, Study protocol

## Abstract

**Background:**

Allergic rhinitis (AR) is a worldwide health problem. Allergen avoidance is strongly recommended for AR patients. Air purification can reduce concentrations of particles in indoor air, including those of allergens. Air purifiers have been recommended by clinicians for AR patients, but few studies have focused on the removal of airborne allergens from home environments. Such studies have been limited by a lack of blinding, small samples, or a failure to measure allergen levels, disease activity, or a combination of these factors. This study investigates the efficacy of a high-efficiency air purifier in reducing disease activity in the homes of AR patients sensitive to the allergens produced by *Artemisia* (mugwort) pollen.

**Methods:**

This is a randomized, double-blind, clinical controlled trial that will test active and inactive versions of an air purifier (Atmosphere®; Amway China). Sixty AR patients sensitive to the allergens produced by *Artemisia* pollen will be assigned randomly to two groups of equal numbers. All patients will undergo a 4-week treatment period and a 4-week observation period. Evaluation will be conducted at baseline (day 0) and on days 7, 14, 21, 28, and 56. The primary outcome measure will be the difference in visual analog scale scores from baseline. Secondary outcomes will be changes from baseline in nasal symptoms, allergy symptom scores, responses to the Rhinoconjunctivitis Quality of Life Questionnaire, Epworth Sleepiness Scale scores, and tolerability scores for the air purifier. Side effects of treatment will be recorded.

**Discussion:**

Reducing exposure to allergens can reduce the risk of conditions such as AR. We hypothesise that AR patients sensitive to the allergens produced by Artemisia pollen will not suffer symptoms in a pollen-free environment. AR patients can remove pollen from their homes using air purifiers, decreasing the risk of symptoms. We expect that our study results will provide reliable evidence for determining the effects of air-purification therapy.

**Trial registration:**

ChiCTR-INR-17012481. (Retrospectively registered 26 August 2017).

## Background

Allergic rhinitis (AR) is a global health problem. The reported incidence of AR is 11.8–46% worldwide [1, 2] and 11.1–19.1% [3, 4] in China. Allergen avoidance is strongly recommended for AR patients. In Japan, a country-wide map of pollen concentrations is published daily so that AR patients can avoid high-concentration areas [5]. Seasonal migration of AR patients is an effective but expensive option. We searched for more convenient and less expensive ways to avoid and mitigate allergen exposure.

Our research team focused on air purification because this strategy can reduce the concentrations of particles in indoor air. In addition, air purifiers can filter particles smaller than 1 μm in diameter [6], and the diameter of mugwort (*Artemisia* spp.) pollen is typically 19–25 μm [7] .Air purifiers are recommended by clinicians for AR patients [8], as they can reduce airborne indoor allergen levels significantly [9]. Few studies, however, have focused on the removal of airborne allergens in home environments. Moreover, those studies have been limited by a lack of blinding, small samples, and a failure to measure allergen levels, disease activity, or a combination of these factors.

### Project aim

Studies have suggested that indoor particle purification may reduce clinical symptoms or improve clinical outcomes, particularly for individuals with allergies [10] .Little robust clinical evidence has been provided, however, for the efficacy of indoor-air purification as a treatment. We sought to investigate, by means of a double-blind, placebo-controlled protocol, the efficacy of a high-efficiency particulate air (HEPA) purifier in reducing disease activity in the homes of AR patients sensitive to the allergens produced by *Artemisia* pollen.

## Methods/Design

### Design

**Fig. 1:**
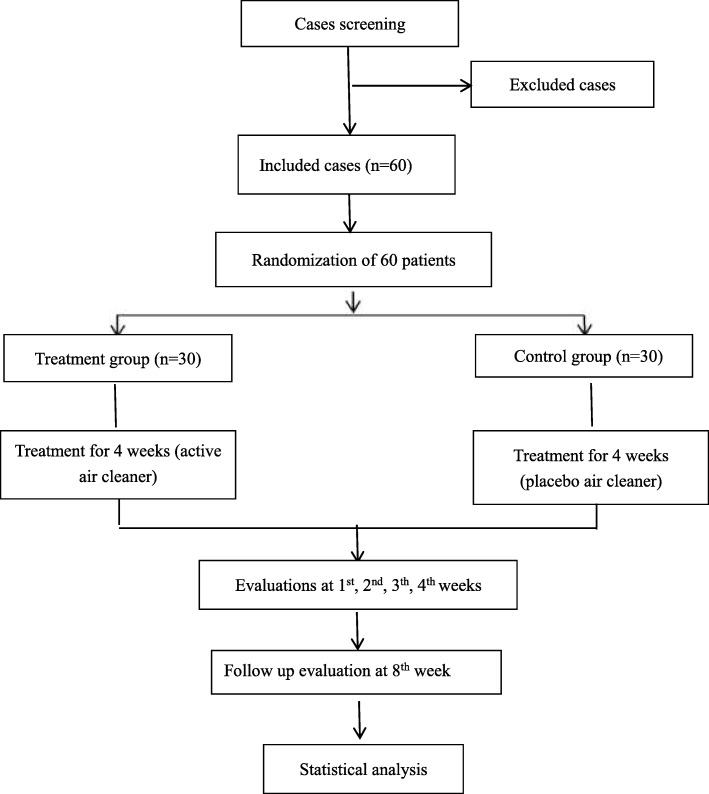
Flowchart of the study design

This is a randomised, double-blind, placebo-controlled clinical trial that tests active and inactive versions of an air purifier. The air purifiers are equipped with monitors to measure the number of hours they operate. AR patients sensitive to the allergens produced by *Artemisia* pollen will be assigned randomly to two groups of equal numbers. All patients will accept a 4-week treatment period and a 4-week observation period. Patient evaluation will be undertaken at baseline (day 0) and on days 7, 14, 21, 28, and 56 (Table 1). Figure 1 displays a flowchart of the study design. Specific tests that will be conducted at each follow-up occasion are outlined in Table 1.

**Table 1:** Schedule of patient evaluation

Outcome measures	Screening stage	Remedial period				Observation period
	Baseline	Day 7	Day 14	Day 21	Day 28	Day 56
Nasal symptoms	X	X	X	X	X	X
Allergy symptom score	X	X	X	X	X	X
Visual analog scale score	X	X	X	X	X	X
Rhinoconjunctivitis Quality of Life Questionnaire	X	X	X	X	X	X
Epworth Sleepiness Scale score	X	X	X	X	X	X
Tolerability of the air purifier		X	X	X	X	
Treatment compliance		X	X	X	X	
Safety assessment		X	X	X	X	X

### Setting

Study activities will be conducted at different settings. Patient enrollment and specimen and data collection will be conducted in the Outpatient Department of the First Hospital of Yulin (Guangxi, China). Biochemical examinations will be undertaken by King Med Diagnostics (Guangzhou, China). Data analyses will be performed at the University of Toronto (Toronto, ON, Canada).

### Study procedures

#### Staff training

Personnel who participate directly in this study will be trained to ensure the safety of patients, blinding of the study design, and data quality.

#### Sample size

The mean changes in findings before and after treatment were used as the indicator for efficacy evaluation in calculation of the sample size. We defined efficacy as changes in at least one score with the visual analog scale (VAS). Using a ratio of 1:1, α = 0.05, and 1 − β = 0.8 with a one-sided *t*-test, we calculated the appropriate trial sample size with 80% power. We found that a clinically significant difference could be detected using a sample of ≥21 patients in each group. We increased this number to 30 per group to allow for a predicted dropout rate of 30%.

#### Recruitment, screening, and enrollment

Clinical recruitment staff will be in charge of enrolling the 60 participants sensitive to the allergens produced by *Artemisia* pollen. We will follow specific criteria to recruit, screen, enroll, and exclude participants. Patients will be enrolled if they meet all inclusion criteria, and will not be enrolled if they meet any of the exclusion criteria, rejection criteria, or termination standards. Participants will be briefed on the purpose, procedures, treatments, and possible risks of the trial. They will be clearly informed of their rights to discontinue participation in this clinical trial. After patients sign consent forms, researchers will assign them to the intervention groups. All of the personal privacy will be protected.

#### Inclusion and exclusion criteria

Inclusion criteria are confirmed AR and sensitivity to allergens from *Artemisia* pollen. Patients must be aged 18–65 years, provide written informed consent, volunteer to participate in this clinical trial, and complete the case report form and other records. Inclusion and exclusion criteria are detailed in Table 2.

**Table 2:** Inclusion and exclusion criteria

Inclusion criteria	Exclusion criteria
Confirmed allergic rhinitis	Mental disorders
Sensitive to *Artemisia* pollen allergens	Systemic disease that the researchers consider to interfere with the study
Aged 18–65 years	Aged under 18 years or over 65 years
Provided informed consent and volunteered to participate in this clinical trial	Not cooperative during examination
Completed the case report form and other records	Employment changes leading to a possible loss to follow-up
	Dysgnosia or behavioral disorders
	The following conditions: nasal polyps, chronic sinusitis, severe nasal deviation, rhinitis medicamentosa, primary sleep disorders (> 1 night/week), obstructive sleep apnea, upper respiratory infection within 2 weeks prior to enrollment, or poorly controlled asthma
	Pregnant or may become pregnant, or lactating with a positive urine pregnancy test
	Drug abuse within the past 3 years
	Must sleep in different bed more than six times in 3 weeks or for more than 3 consecutive nights
	Smoked within the past 1 year
	Sensitive to indoor allergens such as dust mites and pet dander
	Other reasons, at the investigator’s discretion
	Refusal to continue the trial because of a poor curative effect
	Refusal to continue the trial for an unspecified reason
	Loss to follow-up because of a change of address or telephone number
	Loss to follow-up because of personal reasons

#### Rejection criteria and termination standards

Rejection refers to excluding patients from the analysis if they (i) do not meet the inclusion criteria; (ii) withdraw written informed consent; (iii) do not receive follow-up care after selection for the trial; (iv) violate the terms of the trial (such as by improper use of the air purifiers, leading to effects that cannot be evaluated).

Termination refers to censoring patients who have met the inclusion criteria but who halted their participation in the trial. Data from these patients will be included in the final statistical analyses. Termination standards include (i) symptoms (e.g., sneezing, runny nose, nasal obstruction, or nose itching) that become severe; (ii) the occurrence of a serious event, or (iii) other health reasons sufficient to halt participation in the study.

#### Consent

Patients who are eligible for the study will be referred by their attending doctor, then the research coordinator will carry out the informed consent process. The research doctor will explain the details of the study to the patients, including its objectives, process, duration, and possible risks and benefits. And patients will sign the consent forms that they are entirely voluntary and their decision to participate in the study will not affect their future medical care. Recruited patients can withdraw from the study at any time for any personal reason. Patients also reserve the right for questioning about the study and the hesitant period. The burden of the intervention assessed will be charged by the research group.

## Follow-up

Upon completion of the 4-week treatment, patients will undergo a 4-week follow-up period. Research staff will continue to follow participants by telephone, short messaging via mobile telephone, or in the clinic. During the treatment period, follow-up will be conducted weekly. A record of symptom assessment and treatment compliance/changes will be maintained. If participants discontinue/deviate from the intervention protocols, research staff will record the reasons for the change. Such participants and their data will be excluded from the study.

### Intervention in the treatment group

The patients will operate an Atmosphere® air purifier (Amway China, Guangzhou, China) in their bedrooms. This air purifier contains a HEPA two-way filter (model number 101076CH) with an airflow velocity of 100/200 cubic feet/minute at a filtration rate of 6000/12,000 cubic feet/hour. This represents 4/8 air changes per hour in a typical bedroom measuring 15 × 12 × 8 ft. The air purifier will be placed in the bedroom of the patient. Instructions will be given for the units to be left running continuously. The patients must stay in their bedrooms at night for 4 weeks (> 8 h each day).

### Intervention in the control group

The patients will operate an Atmosphere air purifier in their bedrooms, but this air purifier will contain a placebo filter. This filter is also of a two-way design with an airflow velocity of 100/200 cubic feet/minute. Instructions will be given for the units to be left running continuously. The patients must stay in their bedroom at night for 4 weeks (> 8 h each day).

### Concomitant care and interventions

During the treatment and observation periods, participants will be prohibited from taking medications such as antihistamines, corticosteroids, decongestants, or leukotriene receptor antagonists orally or intranasally. Only patients with severe symptoms would be permitted to use anti-allergy agents. The type of medication, dose, and use will be recorded by the patient for analysis. For other complex chronic diseases, patients must continue taking their routine medications and therapies. Research staff will record the details of the diseases, medications, and therapies in the case report.

### Primary outcome measure

The visual analog scale (VAS) will be used to measure symptom severity and quality of life. The primary outcome measure is a difference in the VAS.

### Secondary outcome measures

The secondary outcomes will be changes in nasal symptoms, allergy symptom scores, Rhinoconjunctivitis Quality of Life Questionnaire (RQLQ) scores, Epworth Sleepiness Scale scores, and tolerability scores for the Atmosphere air purifier.

#### Nasal symptoms

The most important symptom is a swelling of the turbinates, which will be graded as 1 (“mild”), 2 (“moderate”), or 3 (“severe”).

#### Allergy symptom scores

Allergy symptom scores will be used to grade symptoms as 0 (“no symptoms”), 1 (“mild symptoms”), 2 (“moderate symptoms”), and 3 (“severe symptoms”). The symptoms graded will be congestion, sneezing, nasal itch, rhinorrhea, eye itch, ear/palate itch, eye redness, and tearing.

#### RQLQ

The RQLQ contains 28 questions covering seven topics (daily life activities, sleep, non-eye/nasal symptoms, practical problems, nasal symptoms, eye symptoms, and emotional status). The questions will be graded on a scale from 0 (“none”) to 6 (“very often/always”).

#### Epworth sleepiness score

The Epworth Sleepiness Score comprises eight questions about the patient’s sleepiness, which are scored from 0 (“none”) to 3 (“probably”).

#### Tolerability of the atmosphere air purifier

Tolerability measure of the Atmosphere air purifier is based on answers to five questions, which will be graded on a scale from 1 (“complete disagreement”) to 5 (“complete agreement”).

### Collection and management of data

Research staff is responsible for data collection. The baseline variables will be age, sex, highest education level achieved, dwelling environment, career, diagnosis, results of allergen examination, disease course, family history, VAS score, nasal symptoms, allergy symptom score, RQLQ score, Epworth Sleepiness Scale score, and tolerability of the Atmosphere air purifier. Participants will be required to record the intake of any medication during this study period.

Monitoring and management of data will be performed by a third party, which will build the study database and program settings. All data will be double-imported into an electronic database by two operators. Identified input errors will be corrected until there are no discrepancies in the database. Data organisation, data coding, range checking for data values, and data conversion to ensure quality will be the responsibility of the statistician.

### Statistical analyses

Statistical analyses will be carried out using SPSS v. 17.0 (IBM, Armonk, NY, USA) in the Clinical Evaluation Center of Southern Medical University (Guangzhou, China). Data will be described as the mean and standard deviation for normally distributed data, median and interquartile range for non-normally distributed data, and frequency and proportion for categorical data. All statistical inferences will be determined using two-sided tests. We will set a significance level of 0.05 and use 95% confidence intervals to measure the uncertainty of the estimate. Efficacy analyses will use last observation carried forward (LOCF) methodology to impute for cases not fully followed up during treatment. Pearson’s χ^2^ test will be used to compare the differences between the dropout rate and the dropout rate attributable to adverse events. Baseline data analyses (two sets) will include demographic indicators and general, primary, and secondary indicators before intervention. Measurement data will be compared using a paired *t*-test. For analyses of efficacy, for quantitative variables, comparisons between groups will be made by repeated measurement variance analysis and covariance analysis. For qualitative variables, comparisons between groups will be made using Pearson’s χ^2^ test, whereas central effects will be tested by a mixed-effects model. For rating variables, comparisons between groups will be made using the Kruskal-Wallis test. In terms of analyses of centre effects, the quantitative indicators will be tested by the general linear method, and the qualitative indicators will be tested by the Cochran-Mantel-Haenszel test methods. A logistic regression model will be used to evaluate and correct the rating variables.

### Trial status

This clinical trial was reviewed by the Ethics Committee of the First Hospital of Yulin in 2016. The first patient was enrolled in 2016. From the middle of 2016 to the end of 2017, 45 patients completed the treatment and observation periods. This clinical trial is expected to be completed at the end of 2018.

### Dissemination plan

Researcher will assign the recruited patients anonymous recruitment numbers and de-identified data-sets will be used to perform all subsequent references and analyses. All of the collected patient information of this study will be kept confidential in compliance to the China Personal Data Protection Act. All the data will kept by the full-time manager. And the data will only be available to the researchers. All study data will be kept for 10 years.

## Discussion

The Atmosphere air purfier has been approved for daily use. The Atmosphere air purifier filter (101076CH) was certified to filter pollen allergens by Allergy UK. Before treatment commences, we will explain to patients in detail the research purpose, methods, and possible risks of the clinical trial, and also inform them explicitly of their right to discontinue their participation. Researchers will accept the research plan, treatment procedure, patients, research schedule, case report forms, and written informed consent forms. After the trial, if the participants are willing to continue treatment, we will provide appropriate therapies. Patients who suffer harm from participation in this clinical trial will be provided a certain amount of economic compensation or free treatment, especially if they suffer adverse events. Any moderate/serious adverse events will be reported to the Ethics Committee of the First Hospital of Yulin.

Reducing exposure to allergens can reduce the risk of conditions such as AR. We hypothesise that AR patients sensitive to the allergens produced by *Artemisia* pollen will not suffer symptoms in a pollen-free environment. AR patients can remove pollen from their homes using air purifiers, decreasing the risk of symptoms.

The city of Yulin is part of Guangxi Province in western China, south of the Mu Us Desert. In recent years, *Artemisia desertorum* Sprengel has been sown extensively in and around Yulin, increasing the prevalence of allergic reaction [11–13]. We therefore selected Yulin as the study site. Morris et al. (2006) recommended that individuals with seasonal AR use indoor air purifiers during the ragweed-pollen season [14]. Stillerman and colleagues conducted a 12-week air-purification treatment in patients with perennial AR [15], and found that nasal congestion, sneezing, runny nose, itchy eyes, tearing, and other symptoms improved, as did quality of life. They therefore suggested that reducing allergen exposure effectively has clinical value and benefits AR patients [15]. Therapy based on reducing allergic sensitisation by avoiding allergen exposure at night may have clinical applications. Considering that plant pollen can be removed by air purification, we selected AR patients sensitive to the allergens of *Artemisia* pollen as our research subjects. The treatment periods in the studies cited above were 1–12 weeks, and the follow-up period was short, which may have resulted in inadequate evaluation of the efficacy of air-purification treatments. The duration of follow-up in our study will be 4 weeks, to ensure an adequate evaluation of treatment efficacy. In addition, those studies were not randomised controlled trials. Hence, we will conduct a double-blind, parallel-design, air purifier and placebo controlled randomised clinical trial. We expect that our study results will provide reliable evidence for determining the effects of air-purification treatment.

## Abbreviations

AR: Allergic rhinitis
HEPA: High-efficiency particulate air
LOCF: Last observation carried forward
RQLQ: Rhinoconjunctivitis Quality of Life Questionnaire
VAS: Visual analog scale
